# 385. Safety and Immunogenicity of a Variant-adapted Bivalent (Original/Omicron BA.4/BA.5) BNT162b2 COVID-19 Vaccine Given as a Booster (Dose 4) to 5- to 11-Year-Old Children Who Previously Received 3 Doses of Original BNT162b2

**DOI:** 10.1093/ofid/ofad500.455

**Published:** 2023-11-27

**Authors:** Grant C Paulsen, Lawrence Sher, Charu Sabharwal, Nicholas Kitchin, Sungeen Hill, Emily Wasserman, Xia Xu, Yvonne A Maldonado, Elizabeth Barnett, Janet A Englund, Emmanuel Walter, Flor M Munoz, Eric Simoes, Kawsar R Talaat, Satoshi Kamidani, Lisa Moyer, Vrunda Parikh, Hua Ma, Xingbin Wang, Kenneth Koury, Annaliesa S Anderson, Kena A Swanson, Alejandra C Gurtman, William C Gruber

**Affiliations:** Cincinnati Children's Hospital Medical Center, Cincinnati, Ohio; Peninsula Research Associates, Rolling Hills Estates, California; Pfizer Inc, Pearl River, New York; Pfizer Inc, Pearl River, New York; Vaccine Research and Development, Pfizer Ltd, Hurley, England, United Kingdom; Vaccine Research and Development, Pfizer Inc, Pearl River, New York; Pfizer Inc, Pearl River, New York; Stanford University, Stanford, California; Boston Medical Center, Boston, Massachusetts; Seattle Children’s Hospital, Seattle, Washington; Duke Human Vaccine Institute, Durham, North Carolina; Baylor College of Medicine, Houston, TX; University of Colorado School of Medicine and Colorado School of Public Health, Denver, CO; Johns Hopkins Bloomberg School of Public Health, Baltimore, MD; Emory University School of Medicine and Children's Healthcare of Atlanta, Atlanta, Georgia; Pfizer Inc, Pearl River, New York; Vaccine Research and Development, Pfizer Inc, Pearl River, New York; Vaccine Research and Development, Pfizer Inc, Pearl River, New York; Vaccine Research and Development, Pfizer Inc, Pearl River, New York; Pfizer, Pearl River, NY; Pfizer, Pearl River, NY; Pfizer, Pearl River, NY; Pfizer, Pearl River, NY; Pfizer, Pearl River, NY

## Abstract

**Background:**

A variant-adapted bivalent BNT162b2 mRNA vaccine (bivalent BNT162b2) comprising original SARS-CoV-2 and Omicron BA.4/BA.5 spike proteins is authorized by the US FDA from 6 months of age as a primary series or as booster doses. We studied whether bivalent BNT162b2 booster generates improved immune responses against Omicron BA.4/BA.5 and ancestral strains and had a comparable safety profile to original BNT162b2 in 5–11-year-olds.

**Methods:**

This substudy is part of a phase 1/2/3 master study (NCT05543616) examining safety and immunogenicity of bivalent BNT162b2 in healthy children. The substudy group reported here is open label and evaluates a fourth dose with bivalent BNT162b2 10 μg (5 µg original; 5 µg BA.4/BA.5) in 5–11-year-olds who previously received 3 original BNT162b2 10 μg doses. Reactogenicity (7 day), and 1 month safety and immunogenicity were assessed. SARS-CoV-2 Omicron BA.4/BA.5 and ancestral strain neutralization titers post dose 4 were descriptive immunogenicity endpoints. The comparator group for immunogenicity assessments included 113 participants from the initial pediatric study (NCT04816643) who received 3 original BNT162b2 10 μg doses and who were matched by age and SARS-CoV-2 infection status.

**Results:**

Of the 113 children who received bivalent BNT162b2, 50% were female, 58% White, and 58% SARS-CoV-2 positive at baseline. Median (range) time from dose 3 of original BNT162b2 to bivalent BNT162b2 was 5.5 (2.6−8.6) months. Bivalent BNT162b2 was well tolerated with mostly mild to moderate reactogenicity; no grade 4 events were observed (**Figure**). No serious adverse events were reported. The safety and tolerability profile was generally consistent with that of original BNT162b2. Bivalent BNT162b2 elicited higher neutralizing titers against Omicron BA.4/BA.5 and similar titers against the ancestral strain 1 month post dose 4 compared with original BNT162b2 at 1 month post dose 3 overall and in those who were SARS-CoV-2 positive (**Table**).

**Figure:**
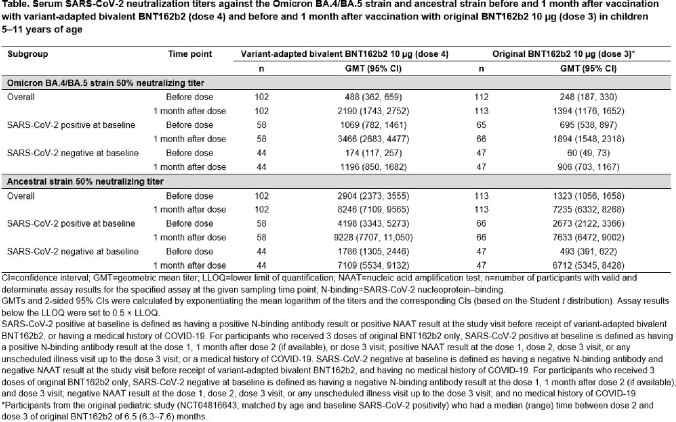

**Figure:**
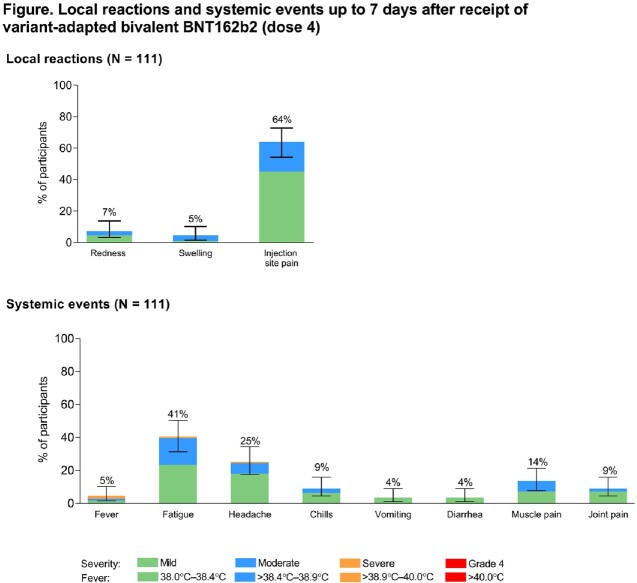

**Conclusion:**

In 5–11-year-olds, a booster (dose 4) of bivalent BNT162b2 10 µg had a similar safety profile to original BNT162b2 10 µg and induced robust Omicron BA.4/BA.5 and ancestral strain neutralizing titers. These data support booster dosing with variant-adapted bivalent BNT162b2 in 5–11-year-olds.

**Disclosures:**

**Grant C. Paulsen, MD**, Moderna: Grant/Research Support|Pfizer: Grant/Research Support **Lawrence Sher, MD**, Pfizer Inc: Clinical Investigator **Charu Sabharwal, MD, MPH**, Pfizer Inc: Employee|Pfizer Inc: Stocks/Bonds **Nicholas Kitchin, MD**, Pfizer Inc: Employee|Pfizer Inc: Stocks/Bonds **Sungeen Hill, MD**, Pfizer Inc: Employee|Pfizer Inc: Stocks/Bonds **Emily Wasserman, MD**, Pfizer Inc: Employee|Pfizer Inc: Stocks/Bonds **Xia Xu, PhD**, Pfizer Inc: Employee|Pfizer Inc: Stocks/Bonds **Yvonne A. Maldonado, MD**, Pfizer: Grant/Research Support|Pfizer: Site Investigator, DSMB member **Janet A. Englund, MD**, Ark Biopharma: Advisor/Consultant|AstraZeneca: Advisor/Consultant|AstraZeneca: Grant/Research Support|GlaxoSmithKline: Grant/Research Support|Meissa Vaccines: Advisor/Consultant|Merck: Grant/Research Support|Moderna: Advisor/Consultant|Moderna: Grant/Research Support|Pfizer: Advisor/Consultant|Pfizer: Grant/Research Support|Sanofi Pasteur: Advisor/Consultant **Emmanuel Walter, MD**, Clinetic: Clinical Investigator|Iliad Biotechnologies: Advisor/Consultant|Moderna: Clinical Investigator|Najit Technologies: Clinical Investigator|Pfizer Inc: Clinical Investigator|Sequiris: Clinical Investigator|Vaxcyte: Advisor/Consultant **Flor M. Munoz, MD, MSc**, CDC respiratory virus surveillance: Grant/Research Support|Gilead: Grant/Research Support|Moderna, sanofi, aztra zeneca, Merck, GSK: Advisor/Consultant|NIH: DSMB|NIH COVID-19 vaccines in pregnancy: Grant/Research Support|Pfizer Pediatric COVID-19 vaccines: Grant/Research Support|Pfizer, Dynavax, Monderna, Meissa, NIH: DSMB **Eric Simoes, MD DCH**, Abbott Diagnostics: Advisor/Consultant|Abbvie Inc: Advisor/Consultant|Abbvie Inc: DSMB study section|AstraZeneca: Grant/Research Support|AstraZeneca: travel|Bill and Melinda Gates Foundation: Advisor/Consultant|Bill and Melinda Gates Foundation: Grant/Research Support|Bill and Melinda Gates Foundation: travel, DSMB study section|CDC: Advisor/Consultant|CDC: travel|GSK plc: Advisor/Consultant|GSK plc: DSMB study section|Johnson & Johnson: Advisor/Consultant|Johnson & Johnson: Grant/Research Support|Merck & Co Inc: Grant/Research Support|National Institutes of Health: Grant/Research Support|National Institutes of Health: travel, DSMB study section|Novavax: Grant/Research Support|Pfizer: Advisor/Consultant|Pfizer: Grant/Research Support|Pfizer: travel|Regeneron: Grant/Research Support|Roche: Grant/Research Support|Roche: travel|USAID: Advisor/Consultant|USAID: Grant/Research Support|USAID: travel|WHO: Advisor/Consultant|WHO: travel **Kawsar R. Talaat, MD**, Intralytix: Advisor/Consultant|Merck: Advisor/Consultant|NIAID: DSMB|Pfizer: Grant/Research Support|Pfizer: Pfizer contract with institution|Sanofi: Grant/Research Support|Takeda: Advisor/Consultant **Satoshi Kamidani, MD**, CDC: Grant/Research Support|Emergent BioSolutions: Grant/Research Support|NIH: Grant/Research Support|Pfizer Inc: Grant/Research Support **Lisa Moyer, BS**, Pfizer: Employee|Pfizer: Stocks/Bonds **Vrunda Parikh, PharmD**, Pfizer: Employee|Pfizer: Stocks/Bonds **Hua Ma, PhD**, Pfizer: Employee|Pfizer: Stocks/Bonds **Xingbin Wang, PhD**, Pfizer: Employee|Pfizer: Stocks/Bonds **Kenneth Koury, PhD**, Pfizer: Employee|Pfizer: Stocks/Bonds **Annaliesa S. Anderson, PhD**, Pfizer: Employee|Pfizer: Stocks/Bonds **Kena A. Swanson, Ph.D.**, Pfizer: Employee|Pfizer: Stocks/Bonds **Alejandra C. Gurtman, M.D.**, Pfizer: Employee|Pfizer: Stocks/Bonds **William C. Gruber, MD**, Pfizer, Inc.: Employee|Pfizer, Inc.: Stocks/Bonds

